# Heterogeneity and effectiveness analysis of COVID-19 prevention and control in major cities in China through time-varying reproduction number estimation

**DOI:** 10.1038/s41598-020-79063-x

**Published:** 2020-12-15

**Authors:** Qing Cheng, Zeyi Liu, Guangquan Cheng, Jincai Huang

**Affiliations:** 1 College of Systems Engineering, National University of Defense Technology, Changsha, 410073 People’s Republic of China; 2 Science and Technology on Information Systems Engineering Laboratory, National University of Defense Technology, Changsha, 410073 People’s Republic of China

**Keywords:** Infectious diseases, Public health

## Abstract

Beginning on December 31, 2019, the large-scale novel coronavirus disease 2019 (COVID-19) emerged in China. Tracking and analysing the heterogeneity and effectiveness of cities’ prevention and control of the COVID-19 epidemic is essential to design and adjust epidemic prevention and control measures. The number of newly confirmed cases in 25 of China’s most-affected cities for the COVID-19 epidemic from January 11 to February 10 was collected. The heterogeneity and effectiveness of these 25 cities’ prevention and control measures for COVID-19 were analysed by using an estimated time-varying reproduction number method and a serial correlation method. The results showed that the effective reproduction number (R) in 25 cities showed a downward trend overall, but there was a significant difference in the R change trends among cities, indicating that there was heterogeneity in the spread and control of COVID-19 in cities. Moreover, the COVID-19 control in 21 of 25 cities was effective, and the risk of infection decreased because their R had dropped below 1 by February 10, 2020. In contrast, the cities of Wuhan, Tianmen, Ezhou and Enshi still had difficulty effectively controlling the COVID-19 epidemic in a short period of time because their R was greater than 1.

## Introduction

On December 31, 2019, the Chinese city of Wuhan reported a confirmed case of the novel coronavirus disease 2019 (COVID-19), and other cities in China also confirmed cases of COVID-19. Consequently, COVID-19 has been spreading to other countries through worldwide air transportation, such as the United States, Spain, Italy, the United Kingdom, Japan, and Thailand^1^, which triggered the global outbreak of COVID-19, similar to those of SARS and Ebola^2^. Because of this worldwide and rapid spread, the World Health Organization (WHO) listed the COVID-19 epidemic as a Public Health Emergency of International Concern (PHEIC) on January 31, 2020^3^. The highly contagious COVID-19 has led to large numbers of infections, health care system overload, and lockdowns in many countries, such as India, Italy and the United Kingdom^4–6^. China was the worst country in the early COVID-19 outbreak, and most Chinese provinces initiated first-level public health emergency responses to the COVID-19 outbreak. For example, Guangdong, Hunan, and Zhejiang provinces initiated a first-level public health emergency response on January 23, 2020, and Hubei, Tianjin, Beijing, Shanghai, Chongqing, Jiangxi, Sichuan, Yunnan, and other provinces also launched first-level public health emergency responses on January 24. In particular, on January 19, 2020, Wuhan, where the COVID-19 epidemic was most severe, implemented strict government policies, including social distancing, extensive testing, and quarantining of confirmed infected subjects to minimize virus transmission via human-to-human contact, and these measures were adopted in other cities^7^. The effectiveness of government policies in slowing the spread of COVID-19 has been discussed by some scholars^8^, and how to measure the effectiveness of the control of the COVID-19 epidemic in each city is the focus of this article.

The effective reproduction number (R) is the expected number of secondary cases generated by an infectious case once an epidemic is underway. R is able to measure the effectiveness of control of the COVID-19 epidemic^9^. There is much research on estimating the R of early COVID-19 propagation^10–12^; for example, the key epidemiologic time-delay distributions and the basic reproduction number were estimated to predict trends of the COVID-19 epidemic in mainland China and provide a theoretical basis for current prevention and control^11–13^. However, the estimated R of the COVID-19 epidemic in these studies is a static value, but R changes dynamically with the prevention and control of the COVID-19 epidemic. Therefore, a time-varying estimate of the effective reproductive number can better quantify the temporal dynamics of the disease^14^, such as the time-dependent reproduction number, which was employed to quantify the temporal dynamics of the disease in African counties and the USA^15,16^. Moreover, previous research focused on the R estimation of the overall transmission of COVID-19, but the epidemic trends in different cities vary considerably due to different policy-making and resource mobilization. This paper aims to answer the following questions. How effective were the control measures taken by cities in China in controlling the COVID-19 epidemic? When will COVID-19 prevention and control measures begin to play an important role in different cities? Is the effectiveness of control of the COVID-19 epidemic the same across cities? A total of 37,726 people were confirmed with COVID-19 in China by February 10, while the confirmed cases in 25 of the worst-hit cities accounted for 92% of the total number. Therefore, based on the number of confirmed cases in the 25 worst-hit cities from January 11, 2020 to February 10, 2020, a dynamic estimation method of R^17^ was used to estimate the R changes in 25 cities to assess the degree of control of the COVID-19 epidemic, and a serial correlation method^18^ was used to analyse the differences in the control effect of the COVID-19 epidemic among cities. Additionally, critical time points for COVID-19 outbreak control in various cities were found by analysing the starting time of R < 1. This study helps to address the effects of COVID-19 control measures in different cities and the differences between cities. It is conducive to improving epidemic prevention measures and providing a reference for epidemic control and prediction of inflection control points^19^. It also provides guidance for the government to adjust prevention and control measures for the COVID-19 epidemic.

## Results

**Figure 1:**
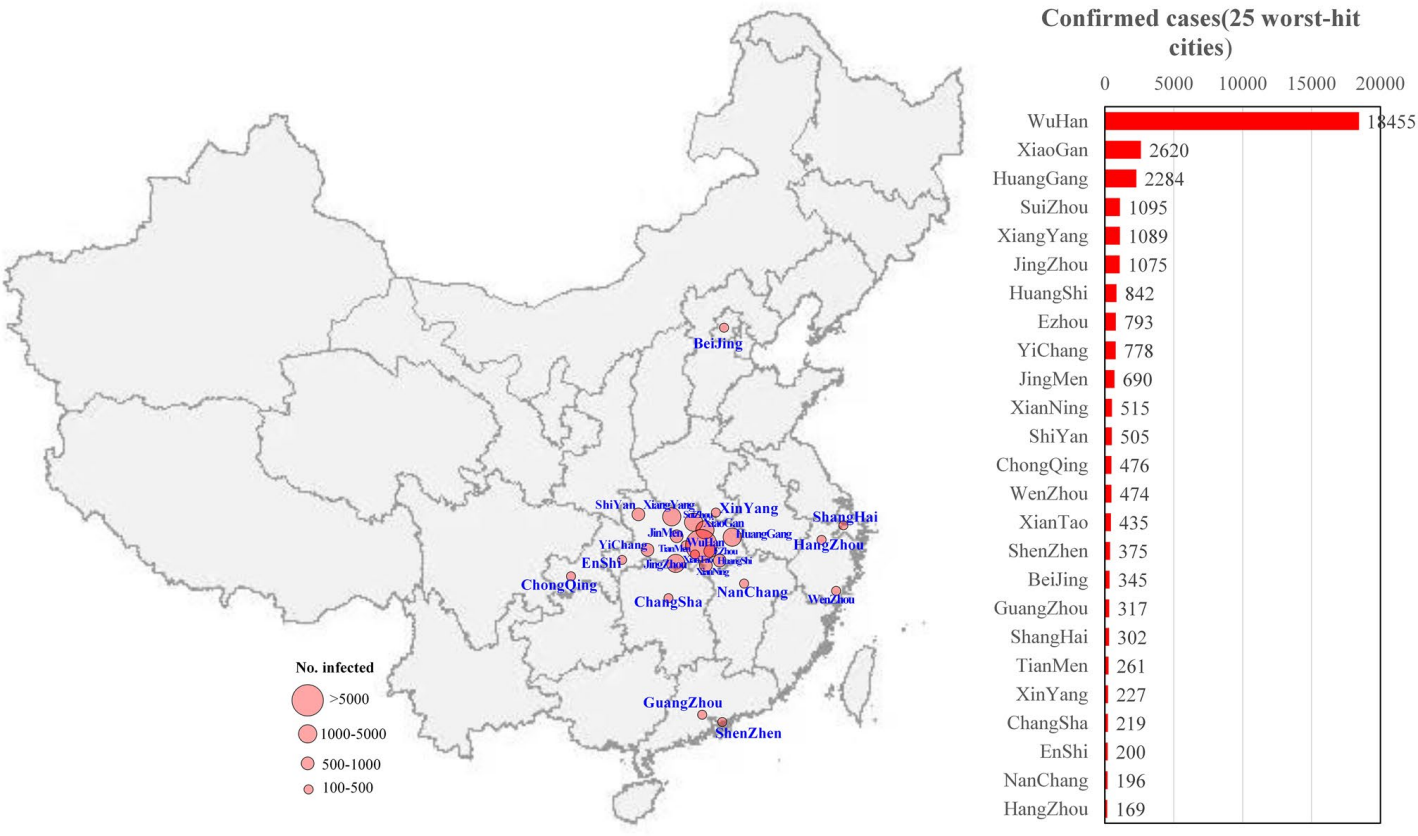
A geographic information map of the cumulative number of confirmed COVID-19 cases as of February 10, 2020 in the 25 worst-hit Chinese cities (the map was created using dituhui.com (www.dituhui.com), and the right panel was created using Excel 2017).

By February 10, 2020, the number of confirmed cases in 25 cities (34,737) accounted for 92% of the total number of confirmed cases in China (37,726). The locations of the 25 cities and the number of confirmed COVID-19 cases are shown in Fig. 1. Most of the worst-hit cities are located in Hubei Province. The worst city was Wuhan (18,455 confirmed cases by February 10), and other cities with cumulative confirmed cases exceeding 1000 included Xiaogan (2620), Huanggang (2284), Suizhou (1095), Xiangyang (1089) and Jingzhou (1075). This distribution is because the source of the outbreak in Wuhan quickly spread to the surrounding cities. Large cities outside Hubei Province with close transport links to Wuhan could also become outbreak epicentres^1^, such as Wenzhou (474), Chongqing (476), Shenzhen (357), Beijing (345), Guangzhou (317), Xinyang (227), Shanghai (302), Changsha (219), Nanchang (196) and Hangzhou (169).

**Figure 2:**
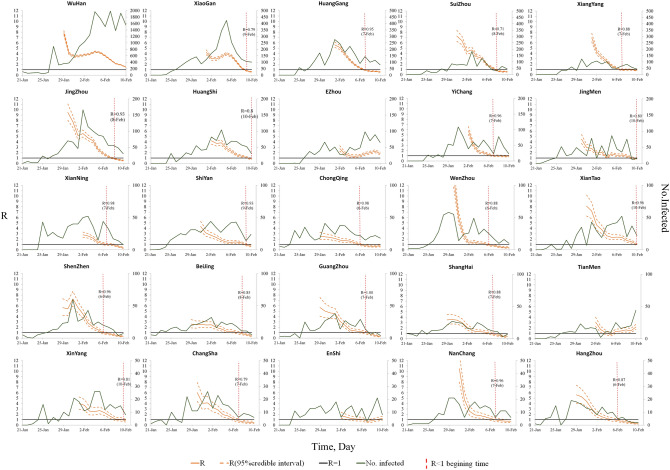
Epidemic curves and R change trends of the 25 worst-hit cities in China for COVID-19.

The R value of the 25 cities was estimated per day by using the time-varying reproduction number estimation method (shown in Fig. 2). All cities’ R values obviously showed an downward trend overall, but there were differences in the R change trends among cities. For example, the R values of Huanggang, Suizhou, Xiangyang, Jinzhou, Huangshi, and other cities declined steadily, while those of Ezhou, Enshi, and Tianmen started to rise after a period of decline. To analyse the heterogeneity of the downward trend in R values among cities, a serial correlation method^18^ was used to calculate the correlation of the R time series among cities.

**Figure 3:**
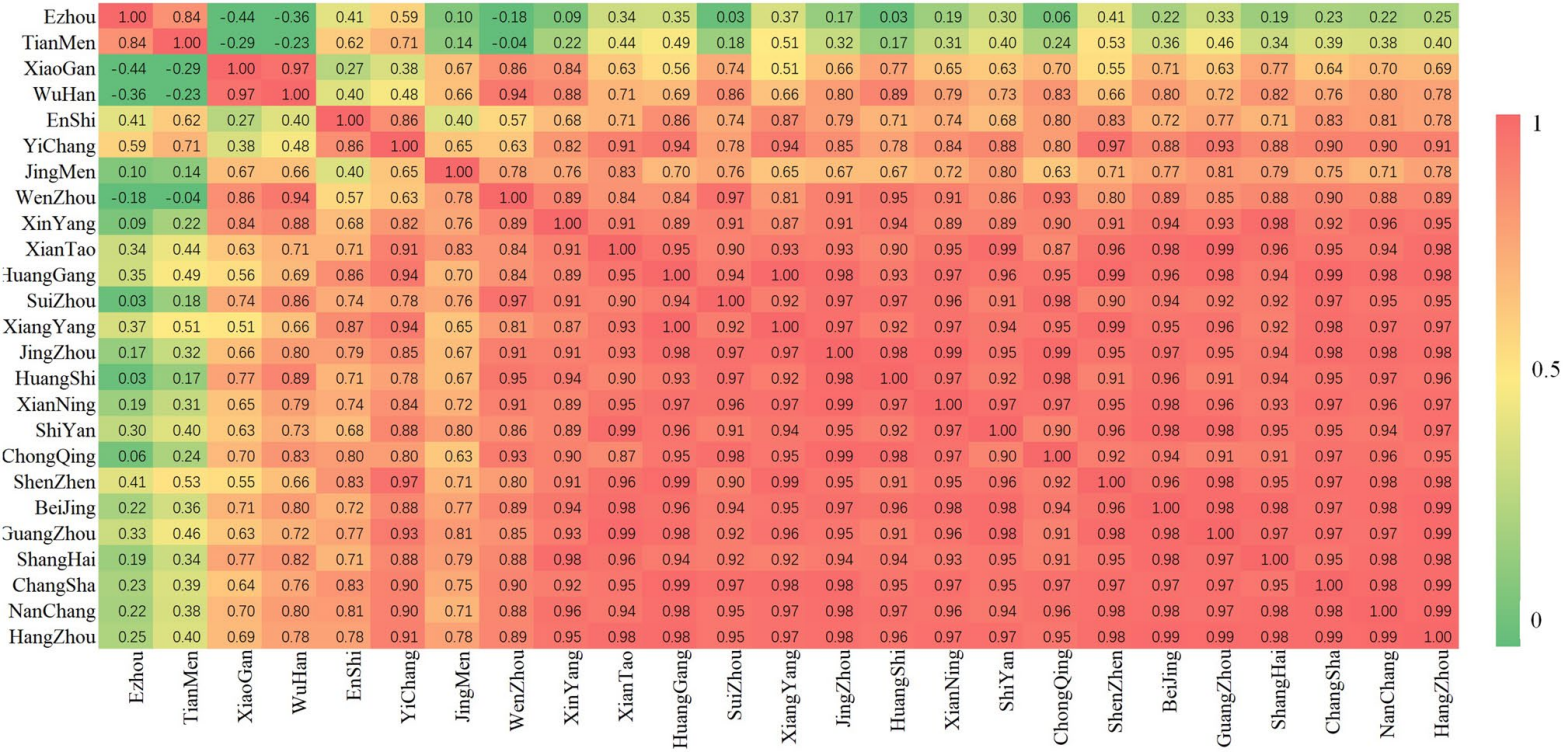
The correlation coefficient of the R time series among cities.

**Figure 4:**
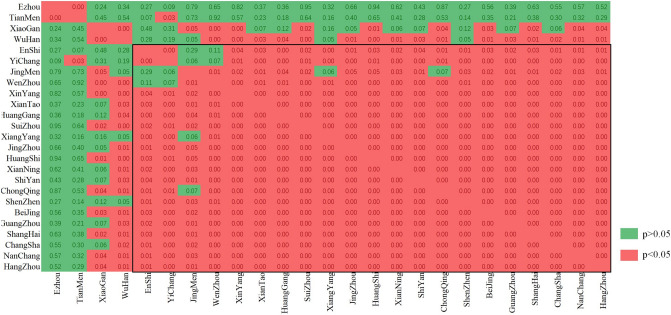
The p value of the correlation coefficient of the R time series among cities (if p < 0.05, the correlation is significant).

The correlation coefficient of the R time series among cities is shown in Figs. 3, and 4 shows the p values for testing the hypothesis of no correlation. From the black box in Fig. 4, it can be seen that except for Ezhou, Tianmen, Xiaogan, and Wuhan, the correlation of the R time series among the other cities was significant. This result shows that although these cities are located in different places, the effectiveness of their COVID-19 control was similar. In addition, after a period of decline, the R values of Ezhou, Tianmen, and Xiaogan slowly increased.

**Figure 5:**
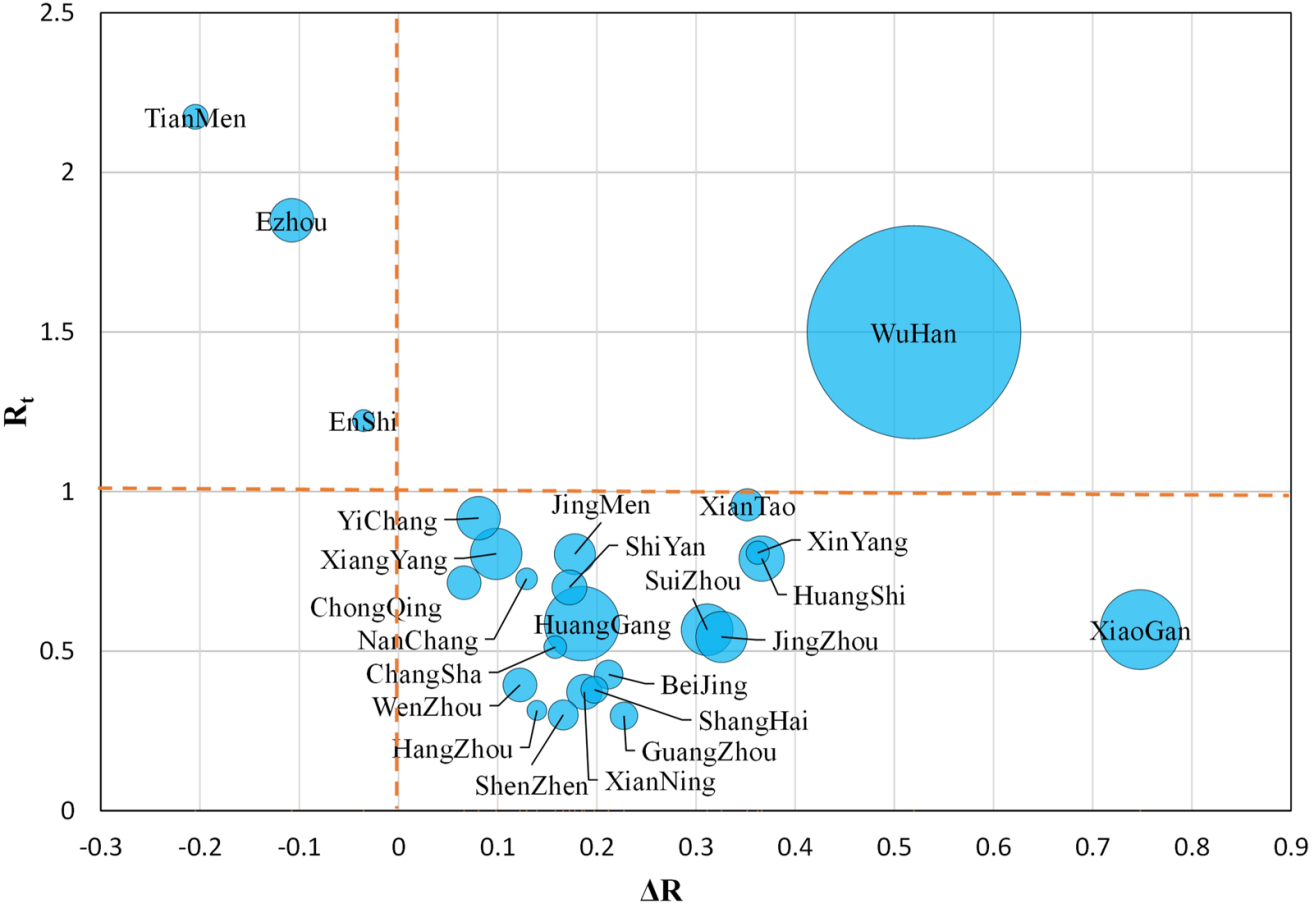
The relationship between the R value on February 10, 2020 (R_t_) and the average decline in R in the past five days (ΔR), where the size of the circle represents the number of confirmed cases in each city.

Although most cities had strong correlations in R change tends, their R values were different. The relationship between the R value of each city on February 10, 2020 (denoted as R_t_) and the average decline in R in the last 5 days (denoted as ΔR) was used to find some patterns, and the result is shown in Fig. 5 (circle size measures the total number of infected cases). In addition, R_t_ = 1 and ΔR = 0 were used to divide Fig. 5. The R_t_ value of the city in the lower right quadrant of Fig. 5 was lower than 1 on February 10, and ΔR > 0 indicated that COVID-19 was effectively controlled in these cities, such as Xiantao, Yichang, Xinyang, Xiangyang, Jingmen, Huangshi, Nanchang, Chongqing, Shiyan, Huanggang, Xiaogan, Shenzhen, and Guangzhou. It is worth noting that all these cities are outside Hubei. For the cities in the upper left quadrant of Fig. 5, not only the R_t_ value is greater than 1 but also ΔR > 0, such as Tianmen (R_t_ = 2.17, 95% CI 1.73–2.67, ΔR = − 0.20), Ezhou (R_t_ = 1.85, 95% CI 1.62–2.10, ΔR = − 0.11), and Enshi (R_t_ = 1.22, 95% CI 0.88–1.62, ΔR = − 0.04). This result showed that these cities not only had a serious COVID-19 epidemic but also had a tendency to continue to deteriorate. As shown in the upper right corner of Fig. 5, Wuhan (R_t_ = 1.50, 95% CI 1.46–1.54, ΔR = 0.52) had a large R value on February 10, although it was consistent with the R trend of most cities. Thus, Wuhan had not yet stabilized control of the COVID-19 epidemic, but the outlook was optimistic, as ΔR > 0.

**Figure 6:**
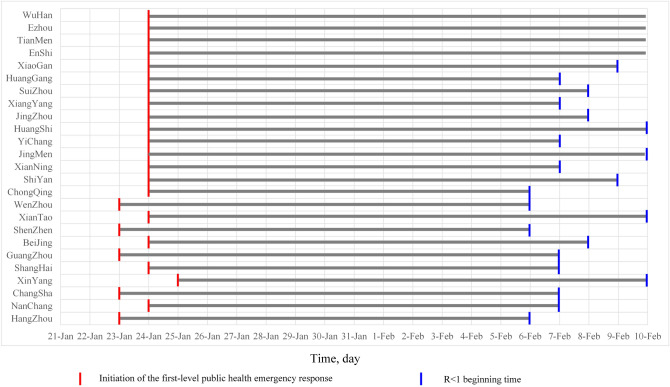
The time at which each city initiated a first-level public health emergency response and the start time for R < 1.

When R drops to 1, the control measures have effectively controlled the epidemic^23^. The starting time of R < 1 was considered the “turning point” for COVID-19 control in our study. It is obvious from Fig. 6 that the starting time of R < 1 was not the same in the various cities. The time at which each city initiated a first-level public health emergency response, how long it took to keep R below 1 and when R continued to decline are shown in Fig. 6. Except for Wuhan, Ezhou, Suizhou, and Tianmen, the cities needed an average of 14.9 days (95% CI 14.4–15.5 days) from the first-level public health emergency response initiated to achieve R < 1 without a significant increase. Thus, the “turning point” of COVID-19 control in most cities is thought to have occurred around February 7.

## Discussion

Calculation and tracking of the R value can effectively monitor the COVID-19 epidemic and judge the effect of the current prevention and control measures. To better implement or modify the COVID-19 prevention and control measures, it is necessary to consider the instantaneous change in R, especially to find a downward trend in the R time series. It is possible to analyse the spatial heterogeneity of COVID-19 transmission by estimating the differences in the change in R among cities. In this paper, 25 of China’s worst-hit cities for the COVID-19 epidemic were selected for R tracking and calculation.

All cities’ R values showed a downward trend overall (Fig. 2), which suggests that the prevention and control measures in these cities played an effective role. However, there was a difference in the R change trend among cities, indicating heterogeneity in the spread and control of COVID-19 in the various cities. Moreover, a major pattern in the R change trend of these cities was found through correlation analysis. Enshi, Yichang, Jingmen, Wenzhou, Xinyang, Xiantao, Huanggang, Suizhou, Xiangyang, Jingzhou, Huangshi, Xianning, Shiyan, Chongqing, Shenzhen, Beijing, Guangzhou, Shanghai, Changsha, Nanchang, and Hangzhou displayed to this pattern, and their R change trends were very similar. We further analysed the R value of these cities on February 10, 2020 and the average decline in R in the past 5 days. The R of these cities (except Enshi) dropped below 1, and ΔR was greater than 0 (the lower right quadrant in Fig. 5), indicating that these cities’ prevention and control measures were effective and that the risk of infection was decreasing. However, the R values of Ezhou, Tianmen, and Enshi were still greater than 1 on February 10, 2020, and the 5-day average decline in R was less than 0. It can be considered that these cities still had difficulty effectively controlling the high risk of COVID-19 in a short period of time, and their R fell for some time and then increased again. The government must continue to strengthen epidemic prevention measures and cannot ease interventions in these cities. It is also worth noting that Wuhan's R was greater than 1, but it was more optimistic that its 5-day average decline was larger.

We compared the time when each city started the first-level public health emergency response and the time when R started to be less than 1. We found that except for in Wuhan, Ezhou, Enshi and Xiaogan, it took approximately two weeks for other cities from start the first-level public health emergency response to effective control of the COVID-19 epidemic. In other words, the “turning point” of COVID-19 control in these cities was considered to occur around February 7, which indicated that the strong measures taken by these cities were effective in containing the epidemic^7^.

The time-varying reproduction number estimation method used in this paper can estimate the real-time trends of the effective reproduction number with joint effects of the random process of personnel mobility and imported cases taken into consideration^21^. However, the local government in Wuhan issued a notice that all within-city and cross-border public transportation was suspended as of January 23, 2020. Since then, the other 12 cities in Hubei Province also implemented similar public traffic control measures, and the interprovincial flow of people was greatly reduced (https://qianxi.baidu.com). The earliest estimate of R was from on January 30, 2020 in the 25 cities in this paper (the start time for R calculation in our model had to fulfil three criteria that will be discussed in detail below). The flow of people between cities was actually very low and had little effect on the reproduction numbers^22^ from January 30, 2020 to February 10, 2020. Therefore, we assumed that the estimation of R did not consider the effect of imported cases. However, it is worth noting that some cities still had imported cases after January 30 because the incubation period of COVID-19 is approximately 14 days or longer^23^. Therefore, the imported cases truly affected the R even after January 30, the estimate of R in our method may be biased^24^. However, because publicly reported data in most cities did not differentiate local cases and imported cases, it is not easy to estimate R by imported cases, so more detailed local and imported case data will be needed to estimate R more accurately.

**Figure 7:**
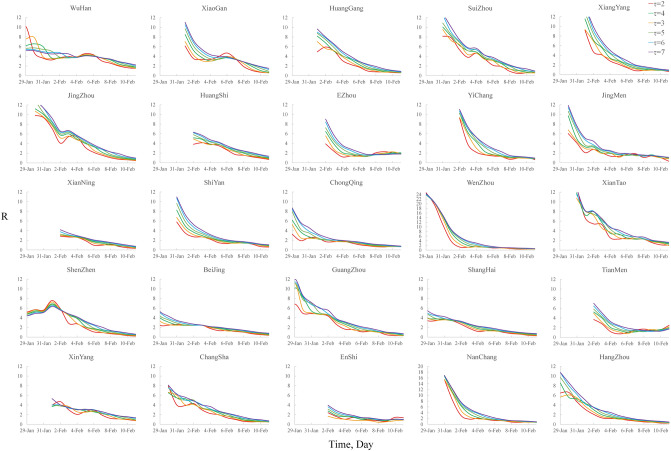
R estimation for 25 cities based on different time windows ($\tau$ = 2, 3, 4, 5, 6, or 7).

On the other hand, the start time for R calculation in our model had to fulfil three criteria^17^. (1) R must be calculated after a time window (time window denoted as $\tau$ and set at 3 in this paper). (2) At least 11 cases must have been observed since the beginning of the epidemic (the time denoted as t_c_) because the minimum number of cases in the time window must satisfy $\sum\nolimits_{s = t - \tau + 1}^{t} {I_{s} \ge \frac{1}{{CV^{2} }} - a}$(*a* = 1, CV = 0.3 in this paper). (3) The estimation of R depends on the probability distribution of the serial interval, that is, it is difficult to observe the complete data to estimate R in a serial interval; thus, an accurate estimation of R must be performed after a serial interval (the average serial interval in this article was 7.5). Therefore, we took the maximum of $\tau$, *t*_c_ and the serial interval as the starting time of the R calculation. Moreover, a smaller time window $\tau$ will result in faster detection of COVID-19 transmission changes, and a larger $\tau$ will result in a smoother estimation. To test the sensitivity of the time window for R estimation, we selected $\tau$ = 2, 3, 4, 5, 6, and 7 and analysed the R estimation in each city. The result is shown in Fig. 7. It can be seen that the R values are slightly different based on different time windows; thus, the selection of different time windows may have an impact on the time when R is first less than 1. However, the changing trend of R will not change, so it will not affect the results of our analysis of the effect of COVID-19 prevention and control in the various cities. To enable faster detection of COVID-19 transmission changes, the minimum reasonable time window ($\tau { = 3}$) was chosen in the studies.

Identifying and tracking changes in R is a way to effectively handle the changes in the epidemic situation in various cities and assist in the guidance of epidemic prevention and control. However, this does not mean that the future R trend of each city will change according to the current development trend. For example, some imported cases from Wuhan may not show symptoms of infection but may be in an incubation period, but they may conceal their contact history and fail to effectively isolate, which may make prevention and control measures invalid and infect more people because it has been proven that COVID-19 can also be transmitted during the incubation period^25^. Therefore, it is necessary to continuously track the number of infections per day and implement an update to calculate the R value. Currently, no city in China can relax epidemic prevention and control measures to end the current epidemic, as imported cases from overseas pose a high risk of a second outbreak^7^.

## Methods and assumptions

### Data collection

The data that support the findings of this study are available from the National Health Commission (http://www.nhc.gov.cn/xcs/yqtb/list_gzbd.shtml) and the Municipal Health Commission of the provinces of the People's Republic of China (see Table S1 in Supplementary Information). The data obtained are publicly available data. After merging COVID-19 data with city-level data by day, the study sample consisted of 34,737 confirmed COVID-19 cases in 25 cities (Beijing, Shanghai, Hangzhou, Wenzhou, Nanchang, Xinyang, Wuhan, Huangshi, Shiyan, Yichang, Xiangyang, Ezhou, Jingmen, Tianmen, Xiaogan, Jingzhou, Huanggang, Xianning, Suizhou, Xiantao, Enshi, Changsha, Guangzhou, Shenzhen, and Chongqing) from January 11, 2020 to February 10, 2020. Cities with a relatively low diagnosis rate were excluded from this analysis. The sample data are described in Table S2.

### Time-varying reproduction number estimation method

We modelled COVID-19 transmission with a Poisson process by assuming the distribution of infectiousness through time after infection is independent of calendar time^17^, so the rate at which someone infected in time step *t*-*s* generates new infections in time step *t* is equal to *R*_t_*w*_s_, where *R*_*t*_ is the instantaneous reproduction number at time *t*, and *w*_s_ is a probability distribution describing the average infectiousness profile after infection. The number infected at time *t* is a Poisson distribution, with $R_{{\text{t}}} \sum\nolimits_{{{\text{s}} = 1}}^{t} {I_{t - s} w_{s} }$ as the mean, *I*_*t-s*_ is the incidences at time step *t-*$\tau$ *s*, and the likelihood of the incidence is *I*_*t*_ given the reproduction number *R*_t_, which is conditional on the previous incidences *I*_*0*_*,…,I*_*t-1*_, is^17^

1 $$P(I_{t} |I_{0} ,...,I_{t - 1} ,w,R_{t} ) = \frac{{(R_{t} \Lambda_{t} )^{{I_{t} }} e^{{ - R_{t} \Lambda_{t} }} }}{{I_{t} !}}$$

where $$\Lambda_{t} = \sum\nolimits_{s = 1}^{t} {I_{t - s} w_{s} }$$.

If the COVID-19 transmission rate is a constant within the time window [*t*- + 1, *t*], which is measured by the reproduction number, it is denoted as $R_{t,\tau }$. The likelihood of the incidence during this time period, $I_{t - \tau + 1} ,...,I_{t}$ given the reproduction number $R_{t,\tau }$, which is conditional on the previous incidences $I_{0} ,...,I_{t - \tau }$, is

2 $$P(I_{t - \tau + 1} ,...,I_{t} |I_{0} ,...,I_{t - \tau } ,w,R_{t,\tau } ) = \prod\limits_{s = t - \tau + 1}^{t} {\frac{{(R_{t,\tau } \Lambda_{s} )^{{I_{s} }} e^{{ - R_{t,\tau } \Lambda_{s} }} }}{{I_{s} !}}}.$$

A Bayesian method using a gamma distribution with parameters (*a*, *b*) is used to represent $R_{t,\tau }$, and the posterior distribution $R_{t,\tau }$ is expressed as

3 $$\begin{aligned} P(I_{t - \tau + 1} ,...,I_{t} ,R_{t,\tau } |I_{0} ,...,I_{t - \tau } ,w) & = P(I_{t - \tau + 1} ,...,I_{t} ,R_{t,\tau } |I_{0} ,...,I_{t - \tau } ,w,R_{t,\tau } )P(R_{t,\tau } ) \\ & = R_{t,\tau }^{{a + \sum\nolimits_{s = t - \tau + 1}^{t} {I_{s} - 1} }} e^{{ - R_{t,\tau } \left( {\sum\nolimits_{s = t - \tau + 1}^{t} {\Lambda_{s} + \frac{1}{b}} } \right)}} \prod\limits_{s = t - \tau + 1}^{t} {\frac{{\Lambda_{s} }}{{I_{s} !}}\frac{1}{{\Gamma (a)b^{a} }}} \\ \end{aligned}.$$

Therefore, the $R_{t,\tau }$ posterior distribution is a gamma distribution with ($a + \sum\nolimits_{s = t - \tau + 1}^{t} {I_{s} } ,\frac{1}{{\frac{1}{b} + \sum\nolimits_{s = t - \tau + 1}^{t} {\Lambda_{s} } }}$) as a parameter. In this paper, we used a gamma prior distribution with parameters *a* = 1 and *b* = 5 to estimate $R_{t,\tau }$. The method was implemented by using the R package “EpiEstim” (http://cran.r-project.org/web/packages/EpiEstim/index.html).

We used the number of confirmed cases per day as the incidence. Therefore, the unknown parameters in the above formula are the time window $\tau$ and *w*_s_. The estimated value of *R*_t_ depends on the choice of the size of the time window $\tau$(assumed $\tau$ = 3 in this paper). According to formula (3), the posterior variation coefficient $R_{t,\tau }$ is ${1/}\sqrt {a + \sum\nolimits_{s = t - \tau + 1}^{t} {I_{s} } }$. Assuming that the posterior coefficient of variation must be less than a threshold CV, the minimum number of cases in the time window must satisfy $\sum\nolimits_{s = t - \tau + 1}^{t} {I_{s} \ge \frac{1}{{CV^{2} }}} - a$. The infectivity profile *w*_s_ can be approximated by the distribution of the serial intervals, and we assumed that the serial interval distribution had a mean (± SD) of 7.5 ± 3.4 days (95% CI 5.3–19 days) based on^11^. Actually^17^, showed that the estimates of R were poorly sensitive to the choice of the prior mean and variance of the serial interval.

In addition, a serial correlation method^18^ was used to calculate the correlation of the R time series among cities. Specifically, the change in the R estimation over time was considered to be a time series of R. Letting the time series of R of two cities be *X* and *Y*, respectively, then, the correlation coefficient of *X* and *Y* was

4 $${\text{co}}rr(X,Y) = \frac{C(X,Y)}{{\sqrt {C(X,X)C(Y,Y)} }}$$

where $C(X,Y) = \frac{{\sum\nolimits_{i = 1}^{n} {(X_{i} - \overline{X})} (Y_{i} - \overline{Y})}}{n - 1}$, and *n* was the length of the time series. In this paper, the latest time to start calculating for all cities’ R was February 10, 2020; thus, the time series of R from February 2 to February 10 was used to calculate the correlation, that is, *n* = 9 in this paper.

## Supplementary Information

Supplementary information

## Data Availability

We confirm that the data obtained is publicly available data, and the data is provided in the Supplementary Information file.
