# Supplementary material for: Heterogeneity and effectiveness analysis of COVID-19 prevention and control in major cities in China through time-varying reproduction number estimation
Source: Sci Rep. 2020 Dec 15;10:21953. doi: 10.1038/s41598-020-79063-x (PMC7738538; doi:10.1038/s41598-020-79063-x)
Supplement: Supplementary file 1 — Supplementary information [file 41598_2020_79063_MOESM1_ESM.docx]

**Heterogeneity and effectiveness analysis of COVID-19 prevention and control in**   **major cities in China through time-varying reproduction number estimation: Supplementary Information**

Qing Cheng^1,2*^, Zeyi Liu^1,2^, Guangquan Cheng^1,2^, Jincai Huang^1,2^

^1^College of Systems Engineering,

National University of Defense Technology,

Changsha 410073, People’s Republic of China

*email:sgggps@163.com

^2^Science and Technology on Information Systems Engineering Laboratory,

National University of Defense Technology,

Changsha 410073, People’s Republic of China

The data that support the findings of our study are available from the Municipal Health Commission of the provinces of the People's Republic of China, the website of Municipal Health Commission of the provinces is shown in Table S1. The number of newly confirmed cases in 25 China’s worst cities for COVID-19 epidemic from January 11 to February 10 was collected (Hangzhou and Wenzhou are located in Zhejiang province, Nanchang is located in Jiangxi province, Xinyang located in Anhui province, Changsha is located in Hunan, Guangzhou and Shenzhen are located in Guangdong, Wuhan, Huangshi, Shiyan, Yichang, Xiangyang, Ezhou, Jingmen, Tianmen, Xiaogan, Jingzhou, Huanggang, Xianning, Suizhou, Xiantao and Enshi are located in Hubei province), the sample data are shown in Table S2.

**Table S1**. the websites of the Health Commission of the provinces of the People's Republic of China

| **Provinecs/cities** | **the website of Health Commission of the provinces** |
| --- | --- |
| Hubei | <http://wjw.hubei.gov.cn/bmdt/ztzl/fkxxgzbdgrfyyq/> |
| Hunan | <http://wjw.hunan.gov.cn/wjw/qlzhyqfkgz/yqfkgz.html> |
| Jiangxi | <http://hc.jiangxi.gov.cn/ztxx/xxgzbdgrdfyyqfk/index.shtml> |
| Zhejiang | <https://wsjkw.zj.gov.cn/> |
| Anhui | <http://wjw.ah.gov.cn/ztzl/xxgzbdfyyqfk/index.html> |
| Guangdong | <http://wsjkw.gd.gov.cn/xxgzbdfk/> |
| Chongqing | <http://cqcdc.org/index.php?a=lists&catid=71> |
| Beijing | <http://wjw.beijing.gov.cn/wjwh/ztzl/xxgzbd/> |
| Shanghai | <http://wsjkw.sh.gov.cn/yqfk2020/> |

| **Time**  **City** | **11-Jan** | **12-Jan** | **13-Jan** | **14-Jan** | **15-Jan** | **16-Jan** | **17-Jan** | **18-Jan** | **19-Jan** | **20-Jan** | **21-Jan** | **22-Jan** | **23-Jan** | **24-Jan** | **25-Jan** | **26-Jan** | **27-Jan** | **28-Jan** | **29-Jan** | **30-Jan** | **31-Jan** | **1-Feb** | **2-Feb** | **3-Feb** | **4-Feb** | **5-Feb** | **6-Feb** | **7-Feb** | **8-Feb** | **9-Feb** | **10-Feb** |
| --- | --- | --- | --- | --- | --- | --- | --- | --- | --- | --- | --- | --- | --- | --- | --- | --- | --- | --- | --- | --- | --- | --- | --- | --- | --- | --- | --- | --- | --- | --- | --- |
| **Beijing** | 0 | 0 | 0 | 0 | 0 | 0 | 0 | 0 | 0 | 5 | 5 | 4 | 12 | 10 | 15 | 17 | 12 | 11 | 20 | 21 | 24 | 27 | 32 | 16 | 25 | 21 | 23 | 18 | 11 | 11 | 5 |
| **Shanghai** | 0 | 0 | 0 | 0 | 0 | 0 | 0 | 0 | 0 | 1 | 8 | 7 | 4 | 13 | 7 | 13 | 13 | 14 | 21 | 27 | 25 | 24 | 16 | 15 | 25 | 21 | 15 | 12 | 11 | 3 | 7 |
| **Hangzhou** | 0 | 0 | 0 | 0 | 0 | 0 | 0 | 0 | 0 | 0 | 1 | 0 | 5 | 6 | 15 | 0 | 5 | 19 | 18 | 16 | 13 | 12 | 8 | 14 | 9 | 10 | 5 | 6 | 3 | 2 | 2 |
| **Wenzhou** | 0 | 0 | 0 | 0 | 0 | 0 | 0 | 0 | 0 | 0 | 2 | 2 | 2 | 4 | 8 | 14 | 28 | 54 | 58 | 55 | 14 | 24 | 26 | 49 | 24 | 32 | 25 | 17 | 10 | 16 | 10 |
| **Nanchang** | 0 | 0 | 0 | 0 | 0 | 0 | 0 | 0 | 0 | 0 | 0 | 1 | 1 | 1 | 1 | 4 | 9 | 8 | 21 | 21 | 16 | 6 | 14 | 18 | 13 | 15 | 13 | 6 | 11 | 11 | 6 |
| **Xinyang** | 0 | 0 | 0 | 0 | 0 | 0 | 0 | 0 | 0 | 0 | 0 | 0 | 1 | 4 | 16 | 1 | 6 | 3 | 10 | 7 | 21 | 18 | 11 | 13 | 26 | 26 | 12 | 16 | 13 | 15 | 8 |
| **Wuhan** | 41 | 0 | 0 | 0 | 0 | 4 | 17 | 59 | 77 | 60 | 107 | 60 | 70 | 77 | 46 | 80 | 892 | 315 | 356 | 378 | 576 | 894 | 1033 | 1242 | 1967 | 1766 | 1501 | 1985 | 1379 | 1921 | 1552 |
| **Huangshi** | 0 | 0 | 0 | 0 | 0 | 0 | 0 | 0 | 0 | 0 | 0 | 0 | 0 | 0 | 31 | 5 | 17 | 33 | 27 | 55 | 41 | 43 | 82 | 71 | 104 | 57 | 69 | 68 | 57 | 52 | 30 |
| **Shiyan** | 0 | 0 | 0 | 0 | 0 | 0 | 0 | 0 | 0 | 0 | 0 | 0 | 1 | 4 | 15 | 20 | 25 | 23 | 31 | 31 | 27 | 35 | 44 | 35 | 27 | 35 | 42 | 43 | 29 | 14 | 24 |
| **Yichang** | 0 | 0 | 0 | 0 | 0 | 0 | 0 | 0 | 0 | 0 | 0 | 0 | 0 | 0 | 19 | 11 | 20 | 12 | 54 | 50 | 109 | 77 | 39 | 60 | 44 | 67 | 47 | 23 | 78 | 45 | 23 |
| **Xiangyang** | 0 | 0 | 0 | 0 | 0 | 0 | 0 | 0 | 0 | 0 | 0 | 0 | 0 | 0 | 2 | 34 | 34 | 61 | 32 | 123 | 61 | 94 | 107 | 84 | 103 | 52 | 51 | 69 | 81 | 57 | 44 |
| **Ezhou** | 0 | 0 | 0 | 0 | 0 | 0 | 0 | 0 | 0 | 0 | 0 | 0 | 0 | 1 | 0 | 19 | 37 | 27 | 39 | 66 | 38 | 51 | 28 | 26 | 50 | 41 | 48 | 98 | 70 | 89 | 65 |
| **Jingmen** | 0 | 0 | 0 | 0 | 0 | 0 | 0 | 0 | 0 | 0 | 0 | 1 | 7 | 13 | 17 | 52 | 24 | 28 | 49 | 36 | 24 | 78 | 16 | 55 | 22 | 86 | 45 | 35 | 75 | 12 | 15 |
| **Tianmen** | 0 | 0 | 0 | 0 | 0 | 0 | 0 | 0 | 0 | 0 | 0 | 0 | 0 | 3 | 2 | 8 | 10 | 11 | 10 | 23 | 15 | 17 | 16 | 2 | 11 | 10 | 25 | 16 | 18 | 20 | 44 |
| **Xiaogan** | 0 | 0 | 0 | 0 | 0 | 0 | 0 | 0 | 0 | 0 | 0 | 0 | 0 | 4 | 29 | 45 | 73 | 101 | 125 | 142 | 87 | 121 | 169 | 202 | 342 | 424 | 255 | 172 | 123 | 105 | 101 |
| **Jingzhou** | 0 | 0 | 0 | 0 | 0 | 0 | 0 | 0 | 0 | 0 | 0 | 6 | 2 | 2 | 23 | 14 | 24 | 30 | 50 | 70 | 66 | 46 | 166 | 114 | 100 | 88 | 84 | 56 | 56 | 48 | 30 |
| **Huanggang** | 0 | 0 | 0 | 0 | 0 | 0 | 0 | 0 | 0 | 12 | 0 | 0 | 0 | 0 | 58 | 32 | 59 | 111 | 172 | 77 | 153 | 276 | 244 | 176 | 223 | 162 | 90 | 144 | 100 | 115 | 80 |
| **Xianning** | 0 | 0 | 0 | 0 | 0 | 0 | 0 | 0 | 0 | 0 | 0 | 0 | 0 | 0 | 43 | 21 | 27 | 21 | 18 | 36 | 40 | 40 | 50 | 52 | 36 | 15 | 44 | 33 | 17 | 14 | 8 |
| **Suizhou** | 0 | 0 | 0 | 0 | 0 | 0 | 0 | 0 | 0 | 0 | 0 | 0 | 0 | 5 | 31 | 16 | 18 | 46 | 27 | 85 | 76 | 80 | 74 | 183 | 65 | 128 | 81 | 38 | 31 | 65 | 46 |
| **Xiantao** | 0 | 0 | 0 | 0 | 0 | 0 | 0 | 0 | 0 | 0 | 0 | 0 | 2 | 8 | 1 | 1 | 15 | 2 | 23 | 35 | 7 | 43 | 29 | 19 | 37 | 40 | 42 | 52 | 20 | 37 | 22 |
| **Enshi** | 0 | 0 | 0 | 0 | 0 | 0 | 0 | 0 | 0 | 0 | 0 | 0 | 0 | 11 | 6 | 8 | 13 | 13 | 15 | 9 | 12 | 18 | 6 | 12 | 15 | 6 | 13 | 3 | 11 | 21 | 8 |
| **Changsha** | 0 | 0 | 0 | 0 | 0 | 0 | 0 | 0 | 0 | 0 | 1 | 3 | 4 | 0 | 10 | 4 | 2 | 22 | 11 | 11 | 18 | 26 | 13 | 23 | 16 | 15 | 11 | 6 | 9 | 8 | 6 |
| **Guangzhou** | 0 | 0 | 0 | 0 | 0 | 0 | 0 | 0 | 0 | 0 | 2 | 3 | 2 | 7 | 0 | 25 | 12 | 12 | 16 | 27 | 31 | 38 | 14 | 27 | 21 | 18 | 29 | 14 | 6 | 9 | 4 |
| **Shenzheng** | 0 | 0 | 0 | 0 | 0 | 0 | 0 | 0 | 0 | 9 | 5 | 1 | 0 | 5 | 7 | 9 | 13 | 14 | 23 | 24 | 60 | 26 | 30 | 43 | 20 | 25 | 20 | 17 | 13 | 4 | 7 |
| **Chongqing** | 0 | 0 | 0 | 0 | 0 | 0 | 0 | 0 | 0 | 0 | 5 | 4 | 8 | 30 | 18 | 35 | 22 | 15 | 18 | 41 | 32 | 24 | 38 | 37 | 29 | 23 | 22 | 15 | 20 | 22 | 18 |

**Table S2**. The number of newly confirmed cases in 25 worst-hit cities for COVID-19 from January 11 to February 10 was collected
